# The human DEAD-box helicase DDX3X as a regulator of mRNA translation

**DOI:** 10.3389/fcell.2022.1033684

**Published:** 2022-10-25

**Authors:** Cathal S. Ryan, Martina Schröder

**Affiliations:** Biology Department, Maynooth University, Maynooth, Ireland

**Keywords:** DEAD-box helicase, DDX3X, DDX3, cellular stress, mRNA translation control, RNA binding protein, posttranscriptional regulation, gene expression regulation

## Abstract

The human DEAD-box protein DDX3X is an RNA remodelling enzyme that has been implicated in various aspects of RNA metabolism. In addition, like many DEAD-box proteins, it has non-conventional functions that are independent of its enzymatic activity, e.g., DDX3X acts as an adaptor molecule in innate immune signalling pathways. DDX3X has been linked to several human diseases. For example, somatic mutations in DDX3X were identified in various human cancers, and *de novo* germline mutations cause a neurodevelopmental condition now termed ‘DDX3X syndrome’. DDX3X is also an important host factor in many different viral infections, where it can have pro-or anti-viral effects depending on the specific virus. The regulation of translation initiation for specific mRNA transcripts is likely a central cellular function of DDX3X, yet many questions regarding its exact targets and mechanisms of action remain unanswered. In this review, we explore the current knowledge about DDX3X’s physiological RNA targets and summarise its interactions with the translation machinery. A role for DDX3X in translational reprogramming during cellular stress is emerging, where it may be involved in the regulation of stress granule formation and in mediating non-canonical translation initiation. Finally, we also discuss the role of DDX3X-mediated translation regulation during viral infections. Dysregulation of DDX3X’s function in mRNA translation likely contributes to its involvement in disease pathophysiology. Thus, a better understanding of its exact mechanisms for regulating translation of specific mRNA targets is important, so that we can potentially develop therapeutic strategies for overcoming the negative effects of its dysregulation.

## 1 Introduction

The human DEAD-box protein 3X (DDX3X) is a multifunctional protein implicated in a variety of biological processes. Paralogue DDX3 genes are located on the X and Y chromosomes, giving rise to two closely related proteins: DDX3X and DDX3Y (Park et al., 1998; Kim et al., 2001; Ditton et al., 2004). DDX3X is expressed in both males and females and can escape X-chromosome inactivation in the latter (Cotton et al., 2015). DDX3Y is expressed only in males and displays some functional redundancy with DDX3X, however it also has a different tissue expression pattern, which may have implications for sex differences in DDX3-related function and disease (Ditton et al., 2004; Venkataramanan et al., 2021). Although its expression was thought to be post-transcriptionally restricted to male germ cells (Ditton et al., 2004), DDX3Y has also been implicated in neurodevelopment and immune cell function, suggesting that it may take part in physiological processes outside of spermatogenesis (Vakilian et al., 2015; Szappanos et al., 2018). In this review, we focus on DDX3X, as it is the better studied of the two paralogues. However, it is possible that DDX3Y makes a significant contribution to overall DDX3 function in males, which should be considered in future studies to more clearly delineate redundancies and separate functions of the two paralogues. It is also possible that DDX3Y contributes to functional effects attributed to DDX3X in some studies discussed here, depending on cell types and the extent of cross-reactivity of research tools. DDX3 orthologues in other organisms include Ded1 in *Saccharomyces cerevisiae* (Sharma et al., 2017) and Belle in *Drosophila melanogaster* (Johnstone et al., 2005), and we include some results relating to these well-studied orthologues in this review.

DDX3X and its orthologues are members of the DEAD-box protein family of RNA remodelling enzymes. As such, DDX3X contains two conserved RecA-like domains that constitute its helicase core (Hogbom et al., 2007), which are flanked by N- and C-terminal extensions unique to DDX3X (Floor et al., 2016). The extended N- and C-termini of DDX3X contain intrinsically disordered regions that confer much of DDX3X’s unique functionality by mediating many different protein-protein interactions and by conferring a propensity for liquid-liquid phase separation (Shih et al., 2008; Saito et al., 2019).

As an RNA helicase, DDX3X hydrolyses ATP to ADP and thereby destabilizes secondary structures of bound RNAs (Sharma et al., 2017; de Bisschop et al., 2019). This RNA remodelling activity allows DDX3X to have numerous functions related to RNA metabolism, being implicated in transcription (Chao et al., 2006; Yang et al., 2019; Saikruang et al., 2022) mRNA export (Yedavalli et al., 2004; Lai et al., 2008; Ajamian et al., 2015), and translation. Of note, DDX3X also has biological functions that do not require its ATPase/helicase activity. For example, it acts as an innate immune signaling adaptor in the RIG-I pathway, where it bridges interactions between various signaling proteins to promote induction of type I interferon (Schröder et al., 2008; Gu et al., 2013; Gu et al., 2017). A similar adaptor function has also been shown for its involvement in the WNT pathway (Cruciat et al., 2013; Dolde et al., 2018). Additionally, DDX3X is a positive regulator of the NLRP3 inflammasome and as such contributes to inflammation and pyroptosis (Samir et al., 2019; Kienes et al., 2021). Thus, DDX3X is a multifunctional protein, and some of its roles fall outside its ability to remodel RNA. Here, we focus on DDX3X’s ability to regulate translation, as this is an intensively investigated aspect of its function that mediates many of its downstream physiological effects.

One driving force behind research into DDX3X is its involvement in a range of diseases. For example, DDX3X is an essential host factor used by a diverse range of viruses, such as HIV-1 (Yedavalli et al., 2004; Soto-Rifo et al., 2013; Frohlich et al., 2016), HCV (Ariumi et al., 2007; Angus et al., 2010; Pène et al., 2015) and SARS-CoV-2 (Ciccosanti et al., 2021; Schmidt et al., 2021), making it an attractive target for the development of broad-spectrum antiviral drugs (Brai et al., 2016; Brai et al., 2019; Brai et al., 2020; Kukhanova et al., 2020; Rao et al., 2021). DDX3X has also been found to play a role in several forms of cancer (He et al., 2018), and dysregulation of its role in translation likely mediates at least some of its oncogenic properties. Thus, DDX3X inhibitors are also being explored as anti-cancer drugs (reviewed in Kukhanova et al., 2020). Finally, DDX3X dysfunction has also been linked to neurological diseases. A range of *de novo* germline DDX3X mutations can cause ‘DDX3X syndrome’, a neurodevelopmental condition presenting with intellectual disability, microcephaly, and other symptoms, which may be attributed at least in part to altered translational regulation (Lennox et al., 2020). DDX3X has also recently been linked to neurodegenerative conditions caused by translation of neurotoxic peptides (Cheng et al., 2019; Linsalata et al., 2019). With such diverse links to disease, DDX3X is considered a promising drug target. However, it is becoming increasingly apparent that DDX3X’s role in homeostatic conditions and disease contexts is complex, and further research to characterise the exact cellular functions of DDX3X is needed.

Here, we review the evidence surrounding DDX3X’s roles in translation regulation, because this is emerging as one of its key cellular roles, and its dysregulation is likely involved in DDX3X-linked disease pathophysiology.

## 2 DDX3X is an RNA binding and remodelling protein

As a DEAD-box protein, DDX3X’s ability to bind RNAs and unwind them is relatively well characterised. However, many questions remain about DDX3X’s exact mode of interaction with its RNA targets, and what distinguishes these RNAs from non-target RNAs.

### 2.1 DDX3X’s interactions with RNA

Several biochemical studies have characterised how DDX3X binds its RNA targets. Binding has been observed for both single-stranded (ss) and double-stranded (ds) RNA, however for dsRNA only if there is either a 3′ or a 5′ overhang (de Bisschop et al., 2019). DDX3X binding to RNA is not dependent on the presence of ATP (Soto-Rifo et al., 2012; Song and Ji, 2019) and it does not recognise specific bases. Instead, DDX3X interacts mainly with the phospho-ribose backbone which it uses to discriminate RNA from DNA (Song and Ji, 2019). Thus, DDX3X displays lax binding specificity for RNA substrates *in vitro*. Yeast Ded1 was shown to be capable of oligomerisation, most commonly as a trimer, wherein two Ded1 molecules bind onto an mRNA to recruit a third molecule which unwinds it (Putnam et al., 2015). DDX3X has also been found to be capable of oligomerisation (de Bisschop et al., 2019), with a recent crystal structure of DDX3X-RNA complexes showing it as a dimer (Song and Ji, 2019).

DDX3X can unwind RNA-RNA and DNA-RNA duplexes *in vitro* in the presence of ATP (Sharma et al., 2017), but not blunt duplexes lacking single-stranded overhangs (Epling et al., 2015). It is unclear whether or to what extent DDX3X can unwind DNA-DNA double-strands, with Garbelli et al. (2011) finding unwinding of 5′ and 3′ DNA-DNA substrates, but Sharma et al. (2017) finding no appreciable unwinding of such a substate. Based on evidence from X-ray crystallographic structures of both DDX3X and the related *D*. *melanogaster* DEAD-box helicase Vasa, (Song and Ji, 2019) suggested a model for DDX3X activity on dsRNA. First, two molecules of DDX3X undergo conformational changes to complex with dsRNA. Next, binding of ATP causes conformational changes resulting in a closed form of the helicase and unwinding of the RNA duplex, probably *via* bending of the RNA (Sengoku et al., 2006). Finally, the closed form of the helicase permits ATP hydrolysis (Sengoku et al., 2006), which results in release of the unwound RNA. RNA binding stimulates DDX3X’s ATP hydrolysis activity, which is often used as a measure of its substrate unwinding activity (Epling et al., 2015). de Bisschop et al. (2019) found that DDX3X’s ATP hydrolysis activity varied depending on the RNA substrate (in the form of truncated HIV-1 genomic RNA (gRNA)) and suggested that RNA structural elements may play a role in stimulating DDX3X’s enzymatic activity.

In addition to unwinding their RNA substrates, DEAD-box helicases can form long-lived interactions with RNA in an ATP-bound state known as “clamping.” In the case of the DEAD-box protein eukaryotic initiation factor (eIF) 4A, clamping allows it to act as a nucleator to establish larger protein complexes on mRNAs (Linder and Jankowsky, 2011). Although to date there is not much direct evidence of clamping in the DDX3X/Ded1 helicase family, their known interactions with a variety of RNA Binding Proteins (RBPs) raises the possibility of them having long-lived interactions with RNA in RBP complexes (Nashchekin et al., 2006; Hilliker et al., 2011). Chen et al. (2020) found that the eIF4A inhibitor Rocaglamide A also binds DDX3X in the cleft between its helicase core and polypurine RNA substrates, which results in a state similar to clamping (although in an ATP-independent manner) and leads to a scanning impediment of the affected mRNAs. Although arising from an exogenous intervention with a small molecule inhibitor, this illustrates the potential for DDX3X to exert inhibitory effects on mRNA translation through long-lived interactions with its targets.

### 2.2 DDX3X’s binding to physiological RNA targets

The studies discussed in Section 2.1 defined DDX3X’s interactions with RNA using synthetic substrates, but do not address DDX3X’s binding to diverse transcripts within the cell. Defining these RNA targets across the transcriptome is important for understanding DDX3X function, as it may exhibit specific binding preferences in cells, which in turn impact downstream effects of translation regulation by DDX3X. To address this, several studies have used Cross-Linking and ImmunoPrecipitation (CLIP) methods coupled with RNAseq (CLIP-seq) to identify DDX3X’s cellular RNA targets and its binding sites on a transcriptome-wide scale. These studies mostly agree on the broad patterns of DDX3X RNA binding in the cell. They show that DDX3X binds a large number of transcripts, with one study identifying over 10,000 bound mRNAs (Oh et al., 2016). This is not unexpected given that its yeast equivalent Ded1 binds virtually all yeast mRNAs (Guenther et al., 2018). Although binding to non-coding RNAs was also observed, DDX3X bound primarily to protein-coding transcripts (Oh et al., 2016; Valentin-Vega et al., 2016; Calviello et al., 2021). Binding was observed mainly in exons as opposed to introns, an indication that DDX3X mainly binds mature mRNAs (Valentin-Vega et al., 2016; Calviello et al., 2021; Gong et al., 2021). Among the non-coding RNAs, DDX3X has been observed to bind all three rRNAs (Oh et al., 2016; Calviello et al., 2021). Although this may be an artefact of the high levels of rRNAs within the cell, DDX3X binding to the 18S rRNA between nucleotides 527 and 553 has been identified as a high-confidence interaction, which may constitute a functionally important direct interaction with the ribosome (Oh et al., 2016; Calviello et al., 2021).

Across CLIP-seq studies, metagenomic profiling has shown the distribution of DDX3X binding sites across mRNAs. The highest number of crosslinks was observed in the 5′ untranslated region (UTR), which was further enriched at the translation start site (Oh et al., 2016; Valentin-Vega et al., 2016; Calviello et al., 2021; Gong et al., 2021). DDX3X has also been shown to bind near the transcription start site (Oh et al., 2016), which may indicate its loading proximal to the 5′cap through its interactions with cap-binding proteins (see Section 3). Enriched binding of DDX3X to 5′ UTRs suggests a role for DDX3X in translation initiation, as destabilising RNA secondary structures in the 5′UTR may facilitate interactions with the translation machinery and/or contribute to ribosomal scanning, as discussed in Section 3. It is interesting to contrast DDX3X’s binding preference for coding mRNA’s 5′UTRs observed in CLIP-seq studies with *in vitro* evidence suggesting that DDX3X binds RNA in a non-specific manner (Soto-Rifo et al., 2012; Song and Ji, 2019). This could suggest that DDX3X binding is influenced by structural features in RNAs or by interactions with other RBPs that were absent from *in vitro* studies. Of note, CLIP studies have so far not found a clear consensus sequence for DDX3X binding (Oh et al., 2016; Valentin-Vega et al., 2016; Calviello et al., 2021). This further supports the idea that DDX3X recognises higher-order structural elements in RNAs rather than sequence motifs; and these might elude traditional motif-searching algorithms. In line with this, Perfetto et al. (2021) showed that translation of both human and *Xenopus tropicalis* Ras-related C3 botulinum toxin substrate 1 (RAC1) mRNAs, which have poor sequence conservation but similar predicted secondary structure, is dependent on DDX3X. Nevertheless, some CLIP studies have identified broad characteristics of DDX3X RNA binding sites, such as a high GC content and complex secondary structure (Oh et al., 2016; Calviello et al., 2021). For example, Calviello et al. (2021) observed enrichment of predicted G-quadruplex (G4) structures upstream of DDX3X binding sites, as well as GC-rich regions resembling Cysteine-Enriched Regulators of Translation (CERT)-motifs downstream of DDX3X crosslinking sites. As discussed in Section 3, DDX3X’s interactions with eIFs may also contribute to the observed binding preferences for 5′UTRs of coding mRNAs. Furthermore, interactions with other RBPs could mediate some of DDX3X’s interactions with specific mRNA targets. For example, heterogenous nuclear Ribonucleoprotein K (hnRNPK) was shown to interact with DDX3X and mediate enhanced translation of JUND mRNA during metabolic stress in pancreatic β-cells, possibly through a C-rich motif in the 3′UTR of the JUND mRNA (Good et al., 2019). DDX3X was also recently shown to mediate Endoplasmic Reticulum (ER) associated translation of secreted growth factors through interactions with signal recognition particle (SRP) proteins. This was mediated *via* the 3′UTRs of growth factor mRNAs and did not require DDX3X’s helicase activity, thus it seems to constitute a distinct mechanism of DDX3X for regulating translation of mRNAs encoding secreted proteins (Chen et al., 2021). Interestingly, both studies suggest that regulation occurs through interactions with 3′UTRs, but open questions remain about the motifs and mechanisms of regulation.

Some CLIP-seq studies investigated whether disease-associated DDX3X mutations affect its RNA binding. Oh et al. (2016) found that DDX3X bearing a medulloblastoma-associated R534H mutation that reduces its catalytic activity had a similar overall binding profile to wild-type DDX3X. Additionally, Valentin-Vega et al. (2016) showed that different medulloblastoma-associated DDX3X mutants (M370R, G302V, G325E) retained their RNA binding ability *in vitro*. Overall, this suggests that these mutations do not drastically change the binding of DDX3X to its RNA substrates, although they impact downstream consequences of binding (see Section 4.2 and Section 4.3.1).

In summary, although some broad characteristics of DDX3X binding patterns have been identified, further investigation into the determinants of DDX3X binding to its physiological RNA targets is needed to better understand its interactions with the transcriptome. Additional biochemical studies to determine which RNA characteristics stimulate DDX3X’s ATPase activity would also be useful, as this is likely relevant for its functional interactions with different physiological targets and can influence the downstream consequences of DDX3X-RNA interactions.

## 3 DDX3X interacts with various components of the translation machinery

Many interactions between DDX3X and eIFs, ribosomal proteins, rRNA and other translation-related factors have been described, opening up numerous possibilities for roles in translation regulation. So far, known interactions mainly link DDX3X to translation initiation, in line with its binding enrichment in 5′UTRs and at start codons. However, the mechanistic contributions of DDX3X to these complexes is not fully understood, and the presence of DDX3X in different translation (pre)-initiation complexes may suggest that it can have several distinct or overlapping roles.

### 3.1 DDX3X’s interactions with cap-binding complexes

DDX3X may already be associated with mRNA when it exits the nucleus, as it is a nucleo-cytoplasmic shuttling protein and has been linked to mRNA export (Frohlich et al., 2016; Brennan et al., 2018). mRNAs that are exported from the nucleus are bound at the 5′cap by the cap-binding complex (CBC) consisting of cap-binding proteins 20 and 80 (CBP-20 and CBP-80) (Ishigaki et al., 2001), and at the 3′ end by poly-A binding protein (PABP) (Gray et al., 2015). DDX3X has been found to interact with the CBPs (Chen et al., 2018) and PABP, the latter being a direct interaction with the core domain of DDX3X (Shih et al., 2012). Cytosolic mRNAs bound by the CBC are subject to a ‘pioneer round’ of translation that allows for quality control of new mRNAs and triggering of nonsense-mediated decay (NMD) should a premature stop codon be detected (Ishigaki et al., 2001; Karousis and Mühlemann, 2019). It is possible that DDX3X plays a role in this mechanism, as it has been identified within NMD protein complexes (Schweingruber et al., 2016) and plant DDX3X orthologues have been implicated in the NMD process (Sulkowska et al., 2020). It is however also possible that the DDX3X-CBP interactions facilitate a form of alternative translation initiation (Chen et al., 2018), as discussed in Section 4.4.3.

**FIGURE 1:**
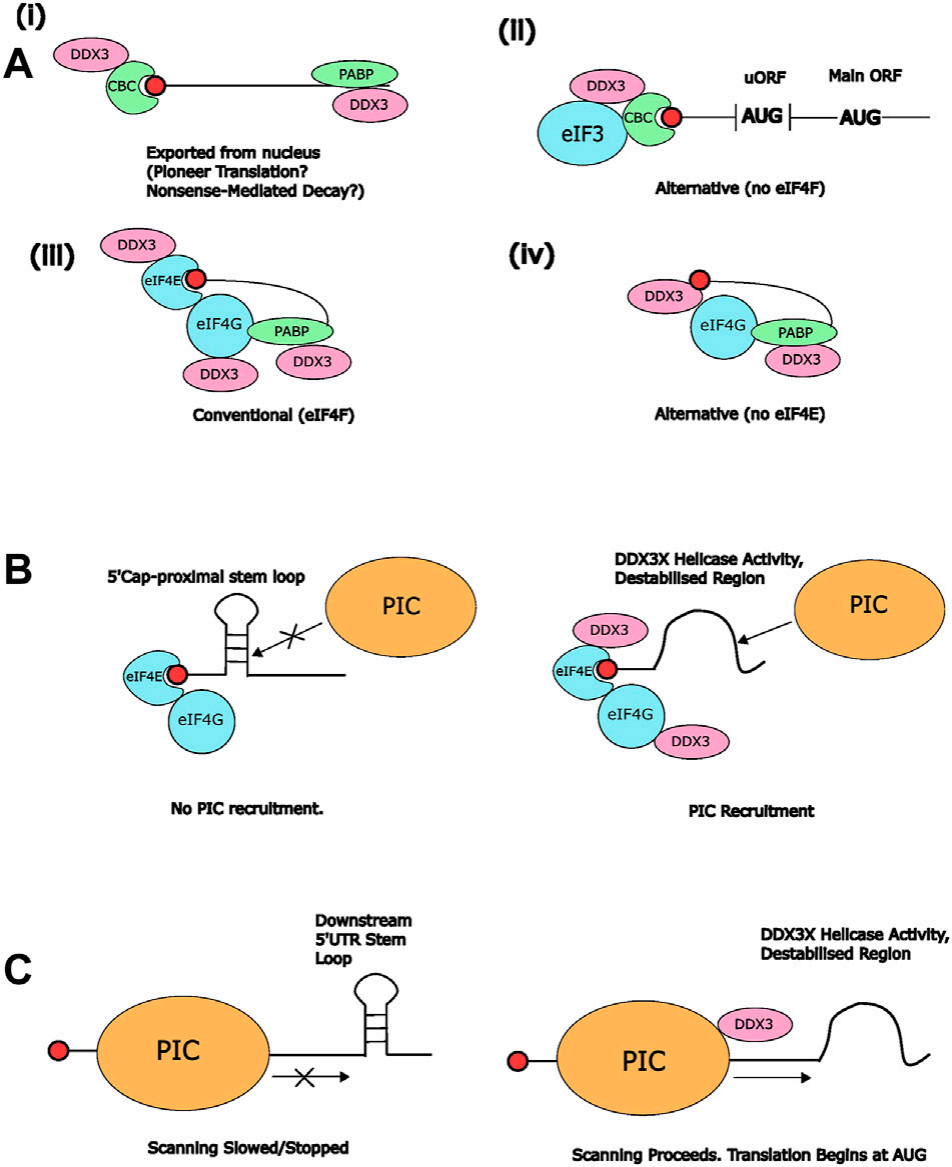
Interactions between DDX3X and other RNA-binding proteins during translation (pre)-initiation. **(A)** Potential DDX3X-containing protein complexes associated with the mRNA 5′cap. Only known DDX3X-interacting factors are included for clarity. (i) DDX3X may be associated with CBC after nuclear export, which could allow it to contribute to pioneer translation and/or nonsense-mediated decay (NMD), or (ii) alternative translation mechanisms independent of eIF4F (Chen et al., 2018). (iii) DDX3X has also been shown to interact with eIF4G (Soto-Rifo et al., 2012) and eIF4E, meaning that it might be associated with the conventional eIF4F complex on RNA (Shih et al., 2008). (iv) Alternatively, it may form novel cap-binding complexes with PABP and eIF4G, but independently of eIF4E (Soto-Rifo et al., 2013) **(B)** Potential contribution of DDX3X helicase activity to recruitment of the 43S PIC to mRNA 5′ends. The presence of stem loops proximal to the 5′ cap can inhibit association of the PIC with the mRNA (left). Through destabilisation of cap-proximal structured regions on the mRNA, DDX3X may support 43S binding (right) (Soto-Rifo et al., 2013). **(C)** Potential contribution of DDX3X to scanning. Structured regions within the 5′UTR downstream of the scanning 48S PIC (left) have the potential to stall scanning and possibly terminate translation initiation (Abaeva et al., 2011 Gupta et al., 2018). Resolution of these structures by DDX3X may facilitate proper scanning of mRNAs containing long, structured 5′UTRs (right).

Following the pioneer round, CBC proteins are usually exchanged for the cap-binding initiation factor eIF4E, which primes the mRNA for further translation by assembling the eIF4F complex. DDX3X has also been shown to directly bind to eIF4E, with a mapped binding site at a 38-YIPPHLR-44 motif in the DDX3X N-terminus, although the C-terminal region also showed some eIF4E-binding ability (Shih et al., 2008). eIF4E associates with eIF4G and A to form the eIF4F complex, and PABP also binds eIF4G to circularise the mRNA, facilitating efficient translation in polysomes (Merrick and Pavitt, 2018). Numerous experiments have shown an interaction between DDX3X and eIF4G (Soto-Rifo et al., 2012; Soto-Rifo et al., 2013; Ayalew et al., 2016; Gong et al., 2021), with direct binding observed between positions 682-1086 of eIF4G (Soto-Rifo et al., 2012). Direct binding to yeast eIF4G, A and E was also observed with Ded1 (Hilliker et al., 2011; Guenther et al., 2018; Gulay et al., 2020), further supporting the notion that DDX3X directly associates with the mRNA-eIF4F complex (Figure 1A).

It is also possible that DDX3X can bind directly to the 5′ methylguanosine cap. Soto-Rifo et al. (2013) found that DDX3X was retained on a 7′mGTP affinity column along with eIF4G and PABP whilst displacing eIF4E, suggesting that it can associate with the 5′cap independent of eIF4E. In line with this, they also found that DDX3X accumulated in cytoplasmic granules containing HIV-1 gRNA, eIF4G, and PABP but not CBP-80 or eIF4E. Based on these results, the authors suggested that DDX3X can substitute for eIF4E in the translation of HIV-1 gRNA. A similar observation was made by Chen et al. (2018) who found that DDX3X favoured recruitment of CBP-20 over eIF4E to the 5′cap of uORF-containing transcripts (Figure 1A, see also Section 4.4.3). In contrast, in experiments conducted by Shih et al. (2008) DDX3X was only observed to bind to a 7′mGTP affinity column when eIF4E was also present. In their experiments, DDX3X reduced the level of eIF4G bound to eIF4E in a dose-dependent manner. The authors proposed that DDX3X disrupts the eIF4E-eIF4G interaction and thereby exerts a negative effect on translation initiation, suggesting that DDX3X can also repress translation. It is possible that the conflicting results can be explained by the different experimental set-ups, as Soto-Rifo et al. (2012) conducted their analysis using HeLa cell extracts whereas Shih et al. (2008) used only purified recombinant proteins and thus other protein co-factors that might modulate the composition of cap-binding complexes were absent in this work.

When recruited to the 5′cap, DDX3X can use its helicase activity to destabilise secondary structures that may otherwise interfere with loading of the eIF4F complex or recruitment of the 43S pre-initiation complex (PIC) (Figure 1B and see Section 3.2) (Soto-Rifo et al., 2013; Shen and Pelletier, 2020). Further studies are required to determine the exact contributions of DDX3X to different cap-binding protein complexes *in vivo*. It is possible that DDX3X’s roles at the 5′cap differ between viral and host translation, or between mRNAs that undergo conventional *versus* alternative translation initiation, and as such are influenced by environmental conditions. Future work should further elucidate whether DDX3X is part of alternative cap-binding complexes in the absence of eIF4E and/or with CBP-20 and how these complexes function. Intriguingly, some studies suggested modulation of DDX3X/Ded1 recruitment and function in cap-binding complexes by (mammalian) Target of Rapamycin (TOR) (Tcherkezian et al., 2014; Nandagopal and Roux, 2015; Aryanpur et al., 2019), and Ded1 appears to be required for growth suppression downstream of TOR inhibition (Aryanpur et al., 2019), suggesting that environmental conditions and cellular stress can alter DDX3X’s recruitment to and role in cap-binding complexes.

### 3.2 DDX3X’s interactions with pre-initiation complexes

The next step in translation pre-initiation involves the association of the 43S pre-initiation complex (consisting of the 40S small ribosomal subunit (SRS), the ternary complex, and eIF3) with the mRNA-eIF4F complex (Merrick and Pavitt, 2018). DDX3X can directly bind to eIF3c (Geissler et al., 2012), and has been observed to associate with numerous other eIF3 subunits (Lee et al., 2008; Chen et al., 2018). Additionally, DDX3X interacts with a variety of 40S ribosomal components (e.g., RPS5, RPS6, RPS11) (Han et al., 2020) and there is evidence of direct binding between DDX3X and the 40S ribosome (Geissler et al., 2012). In addition to binding ribosomal proteins, DDX3X has been found to bind 18S rRNA at a site mapping to helix 16, which faces incoming mRNA on the small ribosomal subunit (Calviello et al., 2021). This binding site has also been observed with Ded1 (Guenther et al., 2018), suggesting that this is an evolutionarily conserved interaction with the pre-initiation complex that allows exposure to mRNAs during the scanning process.

The presence of DDX3X within both the mRNA-eIF4F complex and the pre-initiation complex raises numerous possibilities for potential function(s) during translation initiation. As discussed in Section 3.1, it may have a role in the assembly of cap-binding complexes that activate the mRNA for interactions with the 43S pre-initiation complex. Because DDX3X also interacts with components of the 43S PIC, such as eIF3 and the 40S ribosomal subunit, it is also conceivable that DDX3X directly mediates 43S PIC recruitment to (structured) mRNAs. There is some evidence for this in yeast, where Ded1 can promote 48S formation with mRNAs that contain cap-proximal stem loops predicted to interfere with PIC loading (Gupta et al., 2018). It is conceivable that this mechanism is conserved for DDX3X given that it interacts with eIF4F in a comparable manner to Ded1. However, two studies found no evidence for a contribution of DDX3X to 48S PIC formation. Abaeva et al. (2011) examined formation of 48S complexes with different configurations of eIFs *in vitro* and found no effect of DDX3X. In another study, knockdown of DDX3X was found to increase rather than decrease the number of 48S complexes (Geissler et al., 2012), suggesting that 48S assembly, at least on a global level, can occur in the absence of DDX3X. It is however possible that DDX3X supports 48S assembly only for specific mRNAs, e.g., those containing secondary structures that impede association with the 43S PIC (Figure 1B). An example of this is the HIV-1 gRNA, where DDX3X has been shown to resolve structures at the 5′cap, presumably to enable association with the 43S PIC. This was also observed for several cellular transcripts that contain similar cap-proximal secondary structures, such as NADH Dehydrogenase Ubiquinone 1 alpha subcomplex subunit 1 (NDUFA1) and Interleukin-18 (IL-18) (Soto-Rifo et al., 2012).

It is also a possibility that DDX3X is merely recruited to cap-binding complexes but then exerts its real function during the process of scanning (Figure 1C). Scanning of 5′UTRs for a viable start codon takes place after the mRNA-eIF4F and 43S PIC complexes combine. Yeast Ded1 has been implicated in this process; it promotes scanning and AUG recognition in transcripts containing stem loops (Figure 1C) (Abaeva et al., 2011). Ded1 was found to enhance 48S formation on mRNAs containing cap-distal stem loops to an even greater degree than cap-proximal stem loops in an ATPase-dependent manner, suggesting that it supports their destabilisation during the scanning process (Gupta et al., 2018). Furthermore, profiling of scanning ribosomes showed that deletion of Ded1 caused a shift away from the AUG codon towards the 5′UTR, indicating a reduced rate of scanning (Sen et al., 2019). Based on CLIP-seq data, Guenther et al. (2018) proposed a model for Ded1 function in which it is gradually recruited to the scanning PIC *via* its interactions with eIFs and rRNA, and resolves secondary structures to accelerate scanning, prevent stalling, and support initiation at the start codon of the protein-coding ORF.

In comparison to Ded1, the role of DDX3X in the scanning process is less certain. Soto-Rifo et al. (2012) argued against a role of DDX3X in the scanning process in the translation of HIV-1 genomic RNA (gRNA) and other highly structured mRNAs, as insertion of an unstructured sequence upstream of the cap-proximal stem loop abolished DDX3X dependency, suggesting that DDX3X activity is needed for earlier stages, such as 43S PIC recruitment to the 5′cap complex. On the other hand, knockdown of DDX3X resulted in a global shift in ribosome occupancy from the coding sequence towards the 5′UTR (Calviello et al., 2021), suggesting a defect in scanning rather than 43S PIC recruitment or other earlier stages of pre-initiation. It is also interesting that medulloblastoma-associated DDX3X mutations caused scanning defects when introduced into Ded1, (Brown et al., 2021), indicating a possible conservation in function between the orthologues.

### 3.3 DDX3X’s interactions with the 80S ribosome

Upon finding a start codon in good sequence context, remodelling of the 48S complex takes place and the 60S large ribosomal subunit joins to form the complete 80S ribosome and initiate translation. DDX3X was found to bind the 60S large ribosomal subunit (LRS), mediated through a direct interaction with ribosomal protein L13 (RPL13) (Han et al., 2020). It also binds to 5.8S rRNA and 28S rRNA, although this might be a less significant interaction than with 18S rRNA (Oh et al., 2016; Calviello et al., 2021). It is possible that DDX3X assists in ribosome assembly through these protein-protein and protein-RNA interactions, as one study found it to be associated with newly formed 80S ribosomes, and loss of DDX3X decreased the number of newly formed ribosomes (Geissler et al., 2012).

### 3.4 Involvement of DDX3X in translation elongation and ribosome recycling

To date, most studies have examined DDX3X’s role in translation initiation and not much is known about DDX3X involvement in other stages of translation. Polysome profiling experiments in mammalian cells found that DDX3X associates mainly with pre-initiation complexes and newly formed 80S ribosomes but is not detected in heavier fractions that represent polysomes engaged in translation elongation (Geissler et al., 2012). Nevertheless, Padmanabhan et al. (2021) have detected Leishmania DDX3 in the polysome fraction and identified perturbations in elongation and ribosome recycling after DDX3 knockdown. CLIP-seq studies have also consistently identified DDX3X binding to both CDSs and 3′UTRs, which may indicate a (transient) association with translating ribosomes, however this is much less pronounced than binding in 5′UTRs (see Section 2.2). Further studies would be required to examine whether roles in elongation and ribosome recycling are conserved in mammalian DDX3X.

In summary, through numerous interactions with components of the translation machinery, DDX3X likely maintains an association with the mRNA throughout translation pre-initiation, initiation, and possibly beyond. This gives it opportunities to regulate translation at a variety of stages, including 5′cap recognition and mRNA activation, 43S PIC recruitment, scanning, and ribosomal subunit joining. Although further detailed analysis is needed, it can be speculated that DDX3X may exert several distinct, possibly independent functions at multiple stages of translation initiation, the exact nature of which may depend on structural features in the specific mRNA transcript and the cellular environment.

## 4 Translation regulation by DDX3X and its physiological consequences

Through the aforementioned CLIP-seq experiments, a growing number of mRNAs are being defined as DDX3X targets in various contexts, such as neurodevelopment and cancer However, for many of the mRNAs that were identified in CLIP studies as DDX3X targets, we have no follow-on studies that analyse their regulation by DDX3X. In this section, we will discuss some of DDX3X’s better described mRNA targets and the (potential) physiological consequences of their regulation.

### 4.1 Examples of DDX3X mRNA targets

Across the available studies, several mRNAs have repeatedly been identified as DDX3X targets, and some studies have investigated mechanisms and downstream physiological consequences of DDX3X-mediated translation regulation of specific mRNAs (for examples of DDX3X targets, see Table 1).

**TABLE 1:** Examples of DDX3X targets in the literature. Abbreviations: PAR-CLIP = Photoactivatable Ribonucleotide-enhanced Crosslinking and Immunoprecipitation. FISH = Fluorescence *in situ* hybridisation. TRAP-seq = Translating Ribosome Affinity Purification - RNAseq. Ribo-seq = Ribosome pulldown - RNAseq. LUC = Luciferase reporter translation assays.

RNA target	Experiments	Observed in	Possible physiological consequences	References
Cyclin E	Polysome Fractionation and Microarray, LUC, PAR-CLIP, Ribo-seq, Western Immunoblotting	HeLa, HCT 116, HEK293	Cell Cycle Regulation	Lai et al. (2016)
				Lai et al. (2013)
				Lai et al. (2010)
				Vellky et al. (2020)
				Calviello et al. (2021)
				Lai et al. (2021)
ATF4	Polysome Profiling, LUC, RIP	SAS OSCC, HeLa, Hep3B	Integrated Stress Response, Cancer Progression	Chen et al. (2018)
				Adjibade et al. (2017)
ODC1	LUC, PAR-CLIP, Ribo-seq	HCT 116, HEK293, HeLa	—	Calviello et al. (2021)
				Lai et al. (2022)
				Lai et al. (2008)
RAC1	Polysome Fractionation and Microarray, *In silico* RNA structure predictiion, PAR-CLIP, Ribo-seq, RIP, Biotin-RNA Pulldown, Polysome profiling, Western Immunoblotting, LUC	HCT 116, HEK293, HeLa, N2A Neurite	Neurological Development, Cancer Cell Migration, Macrophage Phagocytosis	Ku et al. (2019)
				Calviello et al. (2021)
				Perfetto et al. (2021)
				Chen et al. (2015)
				Chen et al. (2016)
				Copsey et al. (2017)
p53	LUC	HCT116	Cancer Cell Development	Chen et al. (2017)
RPL12	Western Immunoblotting (iCLAE)	HEK293	Ribosome Biogenesis	Varshney et al. (2021)
RPS12	Western Immunoblotting (iCLAE)	HEK293	Ribosome Biogenesis	Varshney et al. (2021)
RPS19	Western Immunoblotting (iCLAE)	HEK293	Ribosome Biogenesis	Varshney et al. (2021)
TGFß	LUC, RIP	HeLa	Immunity	Lai et al. (2008)
Androgen Recepror	RIP, Western Immunoblotting	BPH1 to cancer progression (BCaP), lymph node carcinoma of the prostate (LNCaP)	Prostate Cancer Resistance to Anti-Androgen Treatment	Vellky et al. (2020)
PACT	Polysome fractionation - microarray, immunoblotting, LUC, *in silico* RNA structure predictiion, CLIP, Ribo-seq	THP-1, HeLa, HCT 116, HEK293	Anti-Viral Innate Immunity	Ku et al. (2019)
				Lai et al. (2016)
				Calviello et al. (2021)
STAT1	Polysome fractionation and microarray, immunoblotting, LUC, *in silico* RNA structure predictiion	THP-1, HeLa	Immunity	Ku et al. (2019)
p38; MAPK	Polysome fractionation and microarray, immunoblotting, LUC, *in silico* RNA structure predictiion	THP-1, HeLa	Immunity	Ku et al. (2019)
TAK1	Polysome fractionation and microarray, immunoblotting, LUC, *in silico* RNA structure predictiion	THP-1, HeLa	Immunity	Ku et al. (2019)
GNB2	Polysome fractionation and microarray, immunoblotting, LUC, *in silico* RNA structure predictiion	THP-1, HeLa	Immunity	Ku et al. (2019)
HSP70	RIP, LUC	HeLa, HEK293	—	Soto-Rifo et al. (2012)
				Geissler et al. (2012)
IL-18	LUC	HeLa	Immunity	Soto-Rifo et al. (2012)
IL-8	LUC	HeLa	Immunity	Soto-Rifo et al. (2012)
Line-1	LUC	HeLa	—	Soto-Rifo et al. (2012)
NDUFA1	LUC	HeLa	—	Soto-Rifo et al. (2012)
JUND	Western Immunoblotting, TRAP-seq	Min6	Metabolic Stress Response	Good et al. (2019)
MITF	RIP, LUC, *in silico* IRES prediction	HT144	Cancer Cell Migration	Phung et al. (2019)
18S; rRNA	CLIP	HEK293	Translation Regulation	Calviello et al. (2021)
				Oh et al. (2016)
HIV-1; gRNA	Florescence Microscopy, Toeprinting, ATPase activity measurements, FISH, Western Immunoblotting	Jurkat, HeLa, HEK293	Viral Infection	Soto-Rifo et al. (2013)
				Lai et al. (2013)
				Soto-Rifo et al. (2012)
				de Bisschop et al. (2019)
				Frohlich et al. (2016)
				Liu et al. (2011)
PKAcα	Western Immunoblotting, Polysome Profiling, LUC	N2A Neurite, HeLa	Neurological Development	Chen et al. (2016)
AREG	Biotinylated RNA pulldown, Ribo-seq, LUC	SAS OSCC	Tumor Microenvironment	Chen et al. (2021)
Rcor2	RIP, Ribo-seq	N2A, Mouse Cortices	Neurological Development	Hoye et al. (2022)
Setd3	RIP, Ribo-seq	N2A, Mouse Cortices	Neurological Development	Hoye et al. (2022)
Topbp1	RIP, Ribo-seq	N2A, Mouse Cortices	Neurological Development	Hoye et al. (2022)
DVL2	LUC, PAR-CLIP, Ribo-seq	HCT 116, HEK293	—	Calviello et al. (2021)
MT-COII	MS Proteomics	MDA-MB-435	Mitochondrial Function	Heerma Van Voss et al. (2017)
MT-NDI	MS Proteomics	MDA-MB-436	Mitochondrial Function and	Heerma Van Voss et al. (2017)
EMCV; gRNA	LUC	Virus	Viral Infection	Su et al. (2018)
				Geissler et al. (2012)
HCV; gRNA	RIP, LUC	Virus	Viral Infection	Su et al. (2018)
				Geissler et al. (2012)
CA16; gRNA	LUC	Virus	Viral Infection	Su et al. (2018)
Echo9; gRNA	LUC	Virus	Viral Infection	Su et al. (2018)
EV71; gRNA	LUC	Virus	Viral Infection	Su et al. (2018)
WNV; gRNA	LUC	Virus	Viral Infection	Geissler et al. (2012)

Cyclin E1 is one of the best studied DDX3X targets, and its 5′UTR has been used as a positive control for assessing DDX3X-mediated translation regulation in subsequent studies (Lai et al., 2013, Lai et al., 2016). Regulation of Cyclin E translation linked DDX3X to cell growth control and G1/S regulation, which carries strong implications for its role in cancer (Lai et al., 2010). Another recurring DDX3X target is Ornithine Decarboxylase 1 (ODC1), which possesses a long, structured 5′UTR that inhibits its translation (Van Steeg et al., 1991). RAC1 is another DDX3X target documented across numerous studies, regulation of which may contribute to DDX3X’s roles in neurological development (Perfetto et al., 2020), cancer cell metastasis (Chen et al., 2015), and macrophage phagocytosis (Ku et al., 2019). Several other DDX3X targets have been linked to cancer, implicating DDX3X in tumorigenesis. For example, DDX3X has been found to regulate both cap- and IRES-dependent translation of the prominent tumour suppressor p53 (Chen et al., 2017). In melanoma cells, DDX3X-mediated regulation of Microphthalmia-associated transcription factor (MITF) translation was shown to be important for the metastatic phenotype (Phung et al., 2019). In prostate cancer cells, androgen receptor (AR) translation is regulated by DDX3, which impacts castration-resistance in this cancer type (Vellky et al., 2020). Interestingly, it is negatively regulated by DDX3, likely *via* sequestration of AR mRNA within stress granules (see Section 4.3.1). Amphiregulin (AREG) is a secreted growth factor whose translation is also regulated by DDX3X, in concert with SRP, to promote translation at the ER in the secretory protein synthesis pathway (Chen et al., 2021) (see also Section 2.2). In addition to RAC1, several other DDX3X targets are involved in neurodevelopment. The 5′UTR of Protein Kinase A (PKA) is similar to that of RAC1, and its translation is also regulated by DDX3X, affecting neurite development (Chen et al., 2016). Through ribosome profiling in a murine cortical development model, Hoye et al. (2022) identified a host of DDX3X-regulated genes involved in neurological development, such as REST Corepressor 2 (Rcor2), SET Domain-Containing Protein 3 (Setd3) and DNA Topoisomerase II Binding Protein 1 (Topbp1). These multiple links to translation of mRNAs for neurodevelopmental genes may provide insights into the causes of DDX3X-associated intellectual disability (DDX3X syndrome) (Hoye et al., 2022). DDX3X also translationally regulates a suite of immune genes, including Transforming Growth Factor ß (TGF-ß) (Lai et al., 2008), IL-8, IL-18 (Soto-Rifo et al., 2012), signal transducer and activator of translation 1 (STAT1), Transforming Growth Factor-Beta-Activated Kinase 1 (TAK1), and Protein Activator of Interferon Induced Protein Kinase EIF2AK2 (PACT) (Ku et al., 2019). The effect on PACT allows DDX3X to indirectly regulate anti-viral innate immune responses, such as interferon ß (IFN-ß) production (Lai et al., 2016). DDX3X also regulates translation of mRNAs encoding mitochondrial proteins, such as Cytochrome C Oxidase II (MT-COII) and NADH Dehydrogenase 1 (MT-NDI), linking DDX3X to energy metabolism (Heerma van Voss et al., 2017). Finally, another important class of DDX3X targets encode proteins involved in translation (Phung et al., 2019), including large and small ribosomal subunit proteins (Varshney et al., 2021), suggesting that DDX3X may indirectly influence translation more broadly (see also Section 4.2).

### 4.2 Does DDX3X contribute to global mRNA translation?

The overall scope of DDX3X’s involvement in mRNA translation is still somewhat controversial, despite numerous studies that have interrogated this aspect of DDX3X function. Some reports characterised it as a general regulator of translation, while others maintained that it is only required for translation of particular mRNA subsets, such as those with highly structured 5′UTRs. This discrepancy is seen across several types of experimental evidence. For example, several studies have analysed effects of DDX3X knockdown on polysome profiles within cells. Most of these studies found that DDX3X knockdown did not affect the overall proportions of polysomes, monosomes, 40S or 60S ribosomal subunits (Lai et al., 2008; Ku et al., 2019; Linsalata et al., 2019), which indicates that global ribosome occupancy and relative rates of initiation/elongation/recycling are not impacted by DDX3X. However, in some studies DDX3X knockdown did reduce the overall proportion of polysomes, suggesting a global defect in translation (Shih et al., 2008; Phung et al., 2019). This is similar to yeast Ded1, where mutations that confer temperature-sensitivity (Sen et al., 2015) and abrogate Ded1-eIF4A/E interactions (Gulay et al., 2020) were found to reduce the polysome/monosome ratio, suggesting a broad role for Ded1 in regulating translation. Conflicting reports also come from studies using 5′UTR luciferase reporter assays as read-outs of DDX3X-mediated translation regulation. Several studies found that DDX3X selectively regulated transcripts with highly structured 5′UTRs and did not affect translation efficiency of reporter mRNAs containing various unstructured 5′UTRs (Soto-Rifo et al., 2012; Çelik et al., 2015; Lai et al., 2021). However, Geissler et al. (2012) observed negative effects on translation of both structured and unstructured 5′UTR mRNA reporters upon DDX3X knockdown. Additionally, studies that assessed global protein synthesis using labelled methionine incorporation are also in disagreement, with some showing no effect of DDX3X knockdown on global translation (Lai et al., 2010; Soto-Rifo et al., 2012), whereas others showed decreases (Gong et al., 2021; Padmanabhan et al., 2021), or even increases (Shih et al., 2008).

Several studies have used ribosome or polysome profiling to characterise transcriptome-wide changes in ribosome/polysome occupancy upon DDX3X or Ded1 manipulation.

In yeast, mutations in Ded1 that confer heat or cold sensitivity repressed translation globally but had more pronounced effects on a select number of mRNAs with long, structured 5′UTRs, consequently dubbed Ded1 hyper-dependent mRNAs (Sen et al., 2015; Guenther et al., 2018; Sen et al., 2021). Therefore, although Ded1 is a general translation factor in yeast, it also specifically supports translation of a distinct subset of mRNAs.

For DDX3X, many studies altered ribosome occupancy only in particular mRNAs, consequently characterised as specific DDX3X targets. Interestingly, generally only a few hundred mRNAs showed changes in ribosome occupancy upon DDX3X manipulation, much fewer than the many 1000s of mRNAs bound by DDX3X in CLIP experiments (Phung et al., 2019; Calviello et al., 2021; Gong et al., 2021). Changes in translation efficiency of these specific transcripts were observed with DDX3X silencing (Phung et al., 2019; Calviello et al., 2021; Chen et al., 2021; Gong et al., 2021), conditional knockout (Hoye et al., 2022), induced DDX3X degradation (Calviello et al., 2021), and expression of disease-associated DDX3X mutants (Oh et al., 2016; Valentin-Vega et al., 2016; Gong et al., 2021). In most studies, inactivation of DDX3X increased translation efficiency of only a few mRNAs, while most targets had decreased translation efficiencies (Phung et al., 2019; Calviello et al., 2021; Gong et al., 2021; Hoye et al., 2022). In contrast, a recent integrated analysis of 80 human heart translatomes found translation efficiency of most DDX3X targets negatively correlated with DDX3X expression levels (Schneider-Lunitz et al., 2021). In addition, higher predicted structural stability of the target mRNA’s 5′UTR was observed in negatively correlating translation efficiency targets (Schneider-Lunitz et al., 2021), contradicting findings from many other studies where DDX3X positively regulated translation of highly structured mRNAs. Of note, this is the only study where DDX3X levels were not manipulated experimentally. Instead, endogenous levels of DDX3X were correlated with translation efficiency of its targets. This may suggest that increased endogenous DDX3X expression can have suppressive effects on target translation; or it could be a tissue-specific difference in DDX3X function. In addition to effects on specific DDX3X target mRNAs, some studies noted global shifts in ribosome occupancy from the CDS to the 5′UTR upon DDX3X knockdown, possibly related to defects in scanning (Valentin-Vega et al., 2016; Calviello et al., 2021) and indicating that DDX3X can affect translation more globally.

We propose two likely explanations for the occasionally observed broad effects of DDX3X manipulation on translation. Firstly, it has emerged in recent years that mRNAs encoding ribosomal proteins are among DDX3X’s specific targets. In CLIP and Ribo-seq studies, gene ontology analyses showed enrichment of terms relating to the translation machinery (Gong et al., 2021). This was also observed in proteomic analyses of DDX3X-depleted cells, suggesting that DDX3X knockdown does indeed reduce expression levels of many ribosomal proteins (Wilky et al., 2016). Two recent studies provided a mechanism for this by demonstrating that DDX3X binds to G-quadruplex regions present in 5′UTRs of mRNAs for several ribosomal proteins; and that this regulates their translation and affects their overall protein levels (Herdy et al., 2018; Varshney et al., 2021). It is therefore conceivable that DDX3X inactivation indirectly affects translation more broadly through regulation of ribosomal protein expression. This can explain conflicting findings in knockdown studies, as transient and short-term DDX3X knockdown would only reveal direct effects on specific DDX3X mRNA targets, whereas sustained silencing may cause indirect effects on ribosome biogenesis that eventually impact global translation. In support of this hypothesis, Gong et al. (2021) found a decrease in global protein synthesis 48 h but not 24 h post-DDX3 silencing.

The second explanation for general effects of DDX3X manipulation on translation relates to its role in stress granule formation discussed in Section 4.3.1. Translation of mRNAs that are sequestered in stress granules is suppressed and it is likely that DDX3X plays a role in shuttling mRNAs into and possibly out of stress granules. As discussed before, DDX3X does associate with a substantial proportion of the protein-coding transcriptome in CLIP-seq studies, but under homeostatic conditions only regulates translation efficiency of a minority of the mRNAs it binds to. Under cellular stress conditions however, DDX3X’s broad mRNA-binding capacity might allow it to sequester a diverse pool of transcripts into stress granules and inhibit their cap-dependent translation. Indeed, the formation of stress granules has been shown to explain the broad suppression of translation observed upon expression of certain medulloblastoma-associated DDX3X mutants, such as G325E, which have reduced enzymatic activity and trigger aberrant stress granule formation in cells (Valentin-Vega et al., 2016). The suppressive effect of DDX3X in this case even extends to non-targets that are sequestered into the DDX3X-induced stress granules by other RBPs (see also Section 4.3.1) Stress granule-mediated effects are most likely to impact studies using ectopic overexpression of DDX3X and/or analysing effects of inactivating DDX3X mutations.

### 4.3 DDX3X regulates translational reprogramming during cellular stress

Stress-induced translational reprogramming allows cells to rapidly respond to altered environmental conditions and to conserve energy by restricting translation to mRNAs necessary for survival and defence (Advani and Ivanov, 2019). At the molecular level, two stress response pathways affect availability of key translation initiation factors. The Integrated Stress Response (ISR) leads to phosphorylation of eIF2α by stress-responsive kinases. This prevents ‘recycling’ of eIF2 during scanning of 5′UTRs and thereby stalls PICs and induces formation of stress granules (Advani and Ivanov, 2019). The second pathway is mediated by mTOR and limits availability of eIF4E through eIF4E-binding proteins (Laplante and Sabatini, 2012). Due to reduced availability of eIF4E and eIF2 during cellular stress, canonical cap- and scanning-dependent translation initiation is suppressed; and mRNAs that are actively translated during these conditions likely rely on non-canonical translation initiation mechanisms. Non-canonical translation initiation mechanisms are diverse and can be mediated for example by Internal Ribosome Entry Sites (IRES), upstream open reading frames (uORFs), cap-independent translation elements (CITEs), G-quadruplexes, or epigenetic modifications to mRNAs (reviewed in Kwan and Thompson, 2019). Much remains to be learned about the exact molecular mechanisms that mediate translation initiation from these different elements, even with respect to their requirements for canonical initiation factors. However, there is increasing evidence that DDX3X is specifically involved in non-canonical translation initiation during cellular stress, which likely contributes to the altered translational responses to stress observed with medulloblastoma-associated DDX3X mutants (Oh et al., 2016; Valentin-Vega et al., 2016).

#### 4.3.1 DDX3X’s role in stress granule formation and function

As mentioned before, regulation of stress granule (SG) formation is another significant process by which DDX3X may affect translation, both globally and for specific target mRNAs. SGs are dynamic liquid-liquid phase separated accumulations of stalled pre-initiation complexes, mRNAs, and various RNA binding-proteins that form in response to cellular stress. Their formation is triggered by disruption of translation and collapse of cellular polysomes, often but not always downstream of eIF2α phosphorylation by stress-sensing kinases (reviewed in Hofmann et al., 2021). SG formation drastically reshapes cellular translation through the sequestering of mRNAs, RBPs, and core translation machinery components. In addition, SGs can also sequester signalling proteins and thereby regulate signalling pathways linked to cell survival and immune defence (Kedersha et al., 2013). When the stress is removed or cells adapt to the stress, disassembly of SGs and resumption of translation can occur. Various protein-protein, RNA-protein, and RNA-RNA interactions are involved in mediating liquid-liquid phase separation of SG components (Hofmann et al., 2021).

Given that it binds many different mRNAs and translation machinery components (see section 2 and Section 3), it is unsurprising that DDX3X is detectable in SGs in response to a variety of stresses (Lai et al., 2008; Shih et al., 2012; Ayalew et al., 2016; Adjibade et al., 2017; Lai et al., 2021). DDX3X incorporation into SGs has been suggested to be dependent on its interaction with eIF4E (see Section 3.1) (Shih et al., 2012). In line with this, recruitment to SGs was shown to be prevented by loss of DDX3X’s N-terminal low complexity domain (amino acid positions 1-115) (Valentin-Vega et al., 2016), which may be due to an abrogated interaction with eIF4E and/or a reduced propensity for liquid-liquid phase separation (Saito et al., 2019). The N-terminus of DDX3X (aa 1-100) was capable of SG induction when ectopically expressed in cells (Shih et al., 2012), further supporting the importance of this region for SG recruitment/formation. Cui et al. (2020) found that treatment with the DDX3X inhibitor RK-33 impaired arsenite-induced SG assembly and suggested that this is due to decreased RNA binding upon loss of DDX3X’s ATP-binding. It is therefore possible that both protein-protein and protein-RNA interactions mediate DDX3X’s involvement in SG formation.

In a similar fashion to many other SG proteins, overexpression of DDX3X can trigger spontaneous SG formation (Shih et al., 2012; Lai et al., 2021). Several disease-associated DDX3X mutations are more prone to triggering spontaneous SGs than wild-type DDX3X, and it is thought that this contributes to disease pathophysiology in medulloblastoma and neurodevelopmental delay (DDX3X syndrome) (Valentin-Vega et al., 2016; Lennox et al., 2020). *Vice versa*, depletion of DDX3X has been reported to prevent SG assembly (Shih et al., 2012; Cui et al., 2020), but not in all cases (Lai et al., 2008; Adjibade et al., 2017), a discrepancy which may be due to DDX3X knockdown efficiency but could also point to a non-essential function for DDX3X in SG formation. More recent studies suggest that DDX3X might have a role in SG maturation but not their initial formation (Jain et al., 2016; Saito et al., 2019).

In being recruited to SGs, DDX3X also sequesters its bound mRNAs, which likely constitutes a form of translational repression by DDX3X (Valentin-Vega et al., 2016; Vellky et al., 2020). As discussed in Section 4.2, DDX3X mutants that trigger spontaneous SG formation induced translational silencing of DDX3X targets and non-targets that was dependent on SGs. Thus, it is likely that DDX3X acts as a broad repressor of canonical translation through its involvement in SG formation under cellular stress conditions and/or when DDX3X’s enzymatic activity is impaired by mutation or other interventions. DDX3X can also repress translation of specific target mRNAs by sequestering them in SGs as has been shown for androgen receptor (AR) mRNA (Vellky et al., 2020).

An interesting question is whether DDX3X can shuttle specific target mRNAs not only into but also out of SGs. The enzymatic cycle of DEAD-box helicases certainly allows them to dynamically modulate its interactions with target mRNAs, e.g., to release sequestered RNAs upon stimulation of its ATP hydrolysis activity. This has been proposed for Ded1 (Hilliker et al., 2011), which was implicated in regulating both the initial response to cellular stress and the recovery phase where translation is resumed (Aryanpur et al., 2019). Similarly, Aditi et al. (2015) found that expression of DDX3X ameliorated aberrant SG dynamics associated with knockdown of Gle1, a protein that can stimulate DDX3X’s ATPase activity. They proposed that DDX3X may interact with Gle1 to aid both assembly and disassembly of SGs in response to heat shock. Saito et al. (2019) showed that posttranslational modifications in the N-terminal intrinsically disordered region (IDR) of DDX3X can regulate its incorporation into SGs. They found that lysine residues within this region are acetylated under homeostatic conditions, which prevents liquid-liquid phase separation. Upon cellular stress, DDX3X was recruited to SGs, where it associated with HDAC6 that deacetylated the lysine residues in DDX3X’s IDR and thereby enhanced its SG association. Deacetylation of DDX3X contributed to SG maturation but not initiation. These examples demonstrate that DDX3X’s function in SGs can be modulated by cellular interaction partners, likely affecting its role in SG dynamics and translational reprogramming.

It is also an interesting question whether all DDX3X-containing granules that were observed in cells are translationally inactive SGs. SGs are notoriously difficult to biochemically characterise, and most studies so far relied on immunofluorescent staining of core SG components. It is likely that there is a diversity of LLP-separated RNP granules with different compositions and functions dependent on the exact cellular conditions. Intriguingly, it was recently discovered that liquid-liquid phase separation of RBPs can also promote translation of bound mRNAs. The RBP Fragile X Mental Retardation Syndrome-Related Protein 1 (FXR1) was shown to actively recruit and promote translation of its target mRNAs after undergoing LLPS (Kang et al., 2022). It is conceivable that DDX3X may possess a similar function. Previously, puromycin-staining of cells expressing R475G mutant DDX3X revealed the presence of DDX3X in granules that co-localised with puromycin-labelled nascent peptide chains, suggesting active translation or stalled polysomes in or near these SG-like bodies (Lennox et al., 2020).

It is also worth considering how DDX3X’s recruitment into SGs affects its involvement in cell signalling pathways. DDX3X has a positive role in NLRP3 inflammasome activation, thus promoting release of the pro-inflammatory cytokine IL-1β and inflammatory cell death through pyroptosis, however, its recruitment into SGs during cellular stress prevents this (Samir et al., 2019). Thus, DDX3X’s recruitment into SGs contributes to cell survival under conditions that can trigger SG formation and NLRP3 inflammasome activation. Previously, it had been shown that recruitment into SGs facilitates an interaction between DDX3X and I kappa B kinase alpha (IKKα) during HCV infection, leading to IKKα activation and downstream upregulation of lipogenesis (Pène et al., 2015). It is currently unknown whether SG localisation also plays a role in DDX3X’s function in the anti-viral RIG-I signalling pathway. However, it is possible that a link exists because RIG-I has been shown to be recruited into so-called anti-viral SGs that promote RNA ligand-binding and downstream signalling (Onomoto et al., 2012).

Overall, DDX3X has many links to stress granule dynamics and the integrated stress response, which may overlap with its role as a regulator of translation.

### 4.4 DDX3 as a mediator of non-canonical translation initiation

During conditions of cellular stress, the translatome is remodelled significantly and non-canonical translation initiation mechanisms become more prevalent, allowing translation of specific mRNAs while canonical cap- and scanning-dependent translation is blocked. DDX3X has been implicated in regulating various forms of non-canonical translation initiation, occurring in different physiological contexts.

#### 4.4.1 DDX3X’s interaction with internal ribosome entry sites (IRES)

As indicated by their name, Internal Ribosome Entry Sites (IRES) mediate direct ribosome recruitment to internal positions in mRNA transcripts and thereby facilitate translation initiation in a cap-independent and, in most cases, scanning-independent manner (Yang and Wang, 2019). IRES are common in viral RNAs where they form highly conserved secondary structures and can be categorised into four classes: class I is able to initiate translation without the need for protein co-factors or initiator tRNA while classes II-IV have an increasing requirement for canonical translation initiation factors and/or additional proteins called IRES-transacting factors (ITAFs) (reviewed in Yang and Wang, 2019). DDX3X has been shown to bind to and support translation initiation from several viral IRES, as discussed further in Section 5. In short, DDX3X promotes translation from IRES contained in 5′UTRs of many positive-sense single-stranded RNA viruses, explaining its role as pro-viral host factor for these viruses. For cellular IRES, DDX3X’s role is less clear, possibly because most cellular IRES-containing transcripts can also be translated in a conventional cap-dependent manner, and because they are not as well defined as viral IRES. Up to 15% of human genes were suggested to contain IRES and to be translated in an IRES-dependent manner during cellular stress (reviewed in Leppek et al., 2018). Some evidence links DDX3X to regulation of such transcripts, where it could conceivably act as an ITAF that mediates eIF and ribosome recruitment or remodels RNA structures, something that is required for many cellular IRES (reviewed in Leppek et al. (2018)). Phung et al. (2019) identified an IRES in the human MITF-M mRNA as a regulatory target of DDX3X when analysing effects of DDX3X knockdown on translation in melanoma cell lines. Translation of this transcript was strongly suppressed in DDX3X knockdown cells, and the authors demonstrated that DDX3X mediates translation of MITF-M in a cap-independent manner *via* a structural element in its 5′UTR that served as an IRES. In their study, DDX3X knockdown also strongly affected translation from the HCV IRES, but not from other cellular IRES, such as those in the p27 and BCL-XL 5′UTRs. This suggests an interesting specificity for DDX3X’s regulation of different IRES, and further research should be conducted to identify the cis and/or trans factors that influence DDX3X’s differential effects on specific viral and cellular IRES. Of note, upstream ORFs and G-quadruplex regions have also been shown to influence cellular IRES activity (reviewed in Leppek et al., 2018), thus effects of DDX3X on these elements (discussed in Section 4.4.2 and Section 4.4.3) could be interlinked with its potential ITAF function.

#### 4.4.2 DDX3 can remodel G-quadruplex regions in mRNA

G-quadruplexes (G4s) are higher-order, stable structures that can be formed by guanine-rich nucleotide sequences in both DNA and RNA. G4s in mRNAs can influence translation efficiency, e.g., in 5′UTRs they impede recruitment of the 43 PIC or ribosome scanning and thereby suppress canonical cap-dependent translation (reviewed in Leppek et al., 2018). G4s can also influence cap-independent translation initiation, exerting either positive or negative effects depending on the specific transcript. For example, it has been shown that G4 structures can act as IRES elements, thus facilitating alternative cap-independent translation of these transcripts (reviewed in Leppek et al., 2018). On the other hand, there are also examples where G4 elements have negative regulatory effects on IRES-mediated translation (Song et al., 2016). G4-binding proteins have been identified, including several DExD/H-box helicases that can unwind G4 structures, such as DHX36, DDX21, and DHX9 (reviewed in Caterino and Paeschke, 2022). When Herdy et al. (2018) conducted an unbiased screen for proteins that bind the G4 element in NRAS mRNA, they identified DDX3X as an additional G4-binding protein. They then identified a wide range of DDX3X binding sites primarily in 5′UTRs that correspond to G4 motifs. They demonstrated that DDX3X interacts with G4s *via* its Glycine-Arginine-rich (GAR) region. In a follow-on study, the authors demonstrated that G4-regions are prevalent in mRNAs encoding ribosomal proteins and that knockdown of DDX3X can suppress synthesis of the encoded ribosomal proteins (Varshney et al., 2021). Similar findings were also published for Ded1, with these studies additionally showing that Ded1 binding destabilises G4s in an ATP-independent manner (Gao et al., 2019; Yan et al., 2021). Regulation of G4-containing transcripts by DDX3X should be a focus of further research, as other studies also identified enriched binding of DDX3X to G-rich regions, and G4s might be linked to DDX3X’s differential effects on RAN and IRES translation. (Section 4.4.1 and Section 4.4.4).

#### 4.4.3 DDX3X’s role in upstream ORF (uORF) translation and initiation site selection

For eukaryotic mRNAs, translation initiation usually begins at the first AUG start codon in the transcript, mediated by ribosomal scanning that starts at the 5′cap. However, it is now known that significant numbers of translation initiation events do not adhere to this rule. Successful initiation depends on the sequence context around the AUG, with strong AUG start codons embedded into a consensus Kozak sequence (Kozak, 1986). AUGs with weaker sequence context can be skipped during ribosomal scanning. Alternative start codon usage can lead to expression of N-terminally truncated or extended protein isoforms compared to the predominant isoform. In many cases however, upstream AUGs do not lead to expression of alternative protein isoforms, but are part of so-called upstream open reading frames (uORFs). uORFs are short open reading frames found upstream of the main protein-coding ORF, sometimes overlapping with it. These are surprisingly prevalent in the human genome; it is now thought that at least 50% of human genes have upstream ORFs (Andreev et al., 2022). While some uORFs express small functional peptides, uORFs are mainly thought to regulate translation efficiency of the protein-coding ORF by preventing re-initiation at the main AUG. Due to this mechanism, most uORF-containing mRNAs have low constitutive translation levels. During cellular stress, when eIF2 levels are limited, ribosomes are more likely to skip the uORF and initiate at the main AUG start codon, leading to increased expression of the encoded protein. The standard example for this type of regulation is Activating Transcription Factor (ATF) 4, a key regulator of the integrated stress response. The ATF4 mRNA contains three uORFs, one of which is overlapping with the main protein-coding ORF. During cellular stress, ATF4 translation is enhanced as initiation occurs more frequently at the AUG for the protein-coding ORF. Chen et al. (2018) demonstrated that DDX3X favours translation of the main ORF in the ATF4 transcript and other select uORF-containing transcripts (ATF5 and DDIT3). Interestingly however, not all uORF-containing transcripts were equally regulated by DDX3X, as CCAAT/Enhancer-Binding Protein Alpha (CEBPA) and ETS Translocation Variant 1 (ETV1) uORF-containing transcripts were unaffected by DDX3X depletion in this study. The authors suggested that DDX3X primarily affects translation initiation in long uORF-containing transcripts, as extension of the short uORFs in CEBPA and ETV1 rendered these transcripts more dependent on DDX3X. The study also demonstrated that presence of DDX3X favoured recruitment of CBP20 rather than eIF4e to the 5′cap of these transcripts, suggesting a possible involvement of the CBC in regulating alternative translation initiation in these uORF-containing transcripts (see also Section 3.1). Adjibade et al. (2017) had also previously demonstrated a positive role for DDX3X in regulating translation of the ATF4 transcript. In contrast to Chen et al. (2018), these authors suggested that DDX3X associates with the classical eIF4F complex (including eIF4E) and regulates translation of the ATF4 transcript through this.

Initiation in uORFs can occur at standard AUG codons, but also at near-cognate codons, such as CUG, the most efficient non-AUG initiation codon (Andreev et al., 2022). DDX3X’s yeast homologue Ded1 was shown to prevent initiation at non-AUG start codons that are immediately upstream of RNA secondary structures. It was suggested that Ded1 accelerates unwinding of these secondary structures and thereby reduces the dwell time of the ribosome. This prevents initiation at the structure-adjacent non-AUG codons, favouring translation of the protein-coding ORF (Guenther et al., 2018). Human DDX3X has also been implicated in regulating initiation from non-AUG codons in the context of repeat-associated non-AUG translation (see Section 4.4.4). In addition, DDX3X’s propensity to suppress translation of non-AUG uORFs was recently suggested to play a role for DDX3X’s contribution to translation of HIV-1 transcripts (Labaronne et al., 2022). Several HIV-1 transcripts contain non-AUG uORFs that reduce translation of the main protein-coding ORF. Transcripts with non-AUG uORFs were particularly sensitive to DDX3X depletion, suggesting that DDX3X prevents translation initiation at the suppressive uORFs to favour translation of the main protein-coding ORF, very much like what has been proposed for Ded1. This may well be relevant for DDX3X’s function as an essential host factor for HIV-1 replication. Interestingly, the study also demonstrated that uORF-encoded peptides stimulated specific T cell responses in HIV-1 infected samples, suggesting that blocking DDX3X activity in HIV-1 infection could suppress viral protein expression and replication while simultaneously enhancing production of antigenic peptides derived from uORFs.

Regulation of translation efficiency in uORF-containing transcripts is influenced by multiple factors, such as sequence context of the initiation codons and adjacent structural elements, spacing and lengths of uORFs, epigenetic modifications of the mRNA (m6A), and transacting factors, such as eIF3 subunits, eIF1 and eIF5, and cap-binding complex (CBC) (reviewed in (Chen and Tarn, 2019)). While structural elements likely play a role, more work is required to elucidate cis and/or trans factors that render a uORF-containing transcript sensitive to DDX3X regulation.

#### 4.4.4 DDX3X regulates repeat-associated non-AUG (RAN) translation

Repeat-associated non-AUG (RAN) translation drives synthesis of toxic aggregating peptides from expanded nucleotide repeat regions associated with several neurodegenerative conditions. Knockdown of DDX3X and its *D. melanogaster* orthologue belle were shown to suppress RAN translation selectively and strongly in transcripts containing expanded CGG repeats from the Fragile X messenger ribonucleoprotein (FMR1) transcript (Linsalata et al., 2019). FMR1 RAN translation causes the neurodegenerative disorder Fragile X-associated tremor/ataxia syndrome (FXTAS). In this instance, the suppressive effect of DDX3X knockdown was unrelated to start codon selection. Instead, it was suggested to be caused by stalling of ribosomes on highly structured GC-rich regions so that these were unable to reach the translation initiation site (Linsalata et al., 2019). In another study, Cheng et al. (2019) also identified DDX3X as a regulator of RAN translation. Their study investigated effects of DDX3X on translation initiation from expanded hexanucleotide GGGGCC repeats in C9ORF72 that are a cause of Amyotrophic Lateral Sclerosis (ALS) and frontotemporal dementia. Interestingly, the authors observed the opposite effect of DDX3X knockdown on RAN translation compared to Linsalata et al. (2019). In this case, knockdown of DDX3X enhanced RAN translation while DDX3X overexpression suppressed it, implicating DDX3X as a negative regulator of RAN-mediated translation for C9ORF72. The authors demonstrated that this was mediated *via* a cap-independent mechanism and suggest that integrity of secondary structure elements (hairpins rather than G4 elements) formed by the GGGGCC repeats is required for RAN translation *via* an IRES-like mechanism. DDX3X’s helicase activity was required for its suppressive effect on RAN translation, and the authors concluded that unwinding of structural elements by DDX3X impedes IRES function of the repeat region, thus blocking translation initiation. In contrast and as discussed earlier, in most instances DDX3X acts as a positive regulator of IRES-mediated translation, as has been shown for several viral and cellular IRES (Section 4.4.1). The two studies on DDX3X’s role in RAN translation illustrate again that DDX3X can have complex and opposing effects on translation, likely linked to the nature and function of secondary structure elements present in specific transcripts. In this context, it is worth noting that the phenomenon of RAN translation was only described in 2011 and as such many mechanistical questions are still unanswered (Zu et al., 2011). It is likely that RAN translation can occur *via* different molecular mechanisms, including cap- and IRES-dependent initiation (Zu et al., 2011; Castelli et al., 2021). Further elucidation of the mechanistic differences between RAN translation occurring in FMR1 and C9ORF72 transcripts may help to explain the observed differential DDX3X effects.

Clarifying the exact contributions of DDX3X to alternative translation initiation mechanisms should be a focus of further studies because it is likely that dysregulation of DDX3X’s role in this process contributes to its involvement in disease.

## 5 DDX3X’s role in viral translation

Viral infection is a disease context in which DDX3X-mediated translation regulation plays a substantial role. DDX3X has both anti-viral functions that are targeted by viral evasion proteins, and pro-viral functions where it is co-opted by viruses to facilitate their life cycles. These roles are not mutually exclusive, i.e., DDX3X recruitment by a virus to support viral replication can simultaneously inhibit its anti-viral function. Anti-viral functions attributed to DDX3X include its capacity to form SGs as previously described (Section 4.3.1), as well as its non-conventional role as an innate immune signalling molecule in the antiviral RIG-I IFN-inducing pathway (Schröder et al., 2008; Gu et al., 2013; Gu et al., 2017; Fullam et al., 2018). Pro-viral functions of DDX3X involve its ability to resolve secondary structures in viral RNAs, its ability to mediate various forms of non-canonical translation initiation (Section 4.4), as well as its ability to recruit translation machinery components (Section 3). Therefore, translation regulation likely underpins many of the proviral effects of DDX3X. As it is being considered a potential target for broad-spectrum antiviral drug development (Brai et al., 2016; Brai et al., 2019; Brai et al., 2020; Kukhanova et al., 2020; Rao et al., 2021), the complexity of DDX3X’s interactions with viruses merits further exploration.

### 5.1 DDX3X interacts with viral mRNAs and supports (cap-independent) translation

DDX3X has been shown to associate with and support translation of a variety of viral RNAs. For example, in Japanese Encephalitis Virus infection, DDX3X is bound by viral replication complex proteins NS3 and NS5 and binds 5′ and 3′UTRs of the viral RNA to support translation and replication (Li et al., 2014). In many viral infections, DDX3X supports viral translation in a cap-independent manner, usually *via* IRES-mediated translation. Cap-independent translation is a common feature of viral RNAs, as it reduces dependency on conventional translation mechanisms which can become downregulated in response to infection (Hoang et al., 2021, and Section 4.4.1 and Section 4.4.2). In Foot and Mouth Disease Virus (FMDV) infection, DDX3X binds to an IRES in the viral RNA together with RPL13 and supports translation *via* recruitment of eIF3e and j (Han et al., 2020). In Enterovirus 71 infection, eIF4G is truncated by viral proteases, and this processed eIF4G binds to DDX3X, presumably to recruit it for unwinding of an inhibitory stem loop within the gRNA IRES (Su et al., 2018). Other IRES that display DDX3X regulation include those present in HCV (Geissler et al., 2012; Pène et al., 2015; Su et al., 2018), encephalomyocarditis virus (EMCV), Echovirus 9, and Coxsackievirus 16, clearly illustrating the extensive contribution of DDX3X-mediated non-canonical translation to viral infections (Su et al., 2018). Additionally, bovine adenovirus pVIII inhibits cap-dependent translation and interacts with DDX3X. By doing so, it prevents DDX3X’s association with capped mRNAs and uses it to recruit various eIFs to the viral RNA (Ayalew et al., 2016).

### 5.2 Viruses manipulate cytosolic granule formation by DDX3X

SG formation is a common cellular response to viral infection and can arise due to interferon signalling from the anti-viral innate immune response (Hoang et al., 2021). As described in Section 4.3.1, SG formation has the effect of sequestering free ribosomes and RBPs that are essential for cap-dependent translation. Therefore, common viral strategies include inhibition of SG formation and usage of cap-independent IRES translation, as described above. Of note, many viruses form replication complexes and sites of translation that resemble SGs. As DDX3X is a known SG factor (Section 4.3.1), it is not surprising that it has also been shown to co-localise in SG-like cytoplasmic foci with various viral RNAs and proteins, although it is often difficult to determine whether these are genuine SGs or another type of RNA granule that promotes viral replication. By recruiting DDX3X, viruses not only acquire an RNA remodelling activity but also benefit from DDX3X’s interactions with translation machinery components that it can recruit to viral RNAs (see Section 3). For example, HCV influences the cellular distribution of DDX3X over the course of infection. Early in infection, an interaction with the HCV RNA 3′UTR causes DDX3X accumulation in SGs with IKKα, Ras GTPase-activating protein-binding protein 1 (G3BP1), and PABP, which leads to IKKα activation and downstream activation of lipogenesis. 24-48 h post-infection, HCV core protein, which directly binds DDX3X (Mamiya and Worman, 1999; Owsianka and Patel, 1999; You et al., 1999), recruits the helicase to lipid droplets (Pène et al., 2015). Herpes Simplex Virus 1 (HSV-1) infection causes DDX3X to aggregate, with some degree of co-localisation with viral particles (Khadivjam et al., 2017). DDX3X is also recruited to viral replication complexes by murine norovirus, which displays highly structured 5′ and 3′ UTRs (Vashist et al., 2012). Many viruses actively inhibit SG formation, and some of these also interact with DDX3X. It is possible that SG induction sequesters DDX3X from sites of viral replication/translation, where it is needed by the virus. Pestivirus is an example of a virus that inhibits SG assembly and recruits DDX3X *via* its viral N-terminal protease (Jefferson et al., 2014). In West Nile Virus (WNV) infection, DDX3X localisation in cytoplasmic granules is disrupted, and it is eventually recruited to perinuclear WNV replication complexes (Chahar et al., 2013). Finally, DDX3X has recently been identified as an important host factor in SARS-CoV-2 infection. It binds to SARS-CoV-2 RNA (Schmidt et al., 2021) as well as its Nucleoprotein, and is recruited to viral replication complexes along with the SG protein G3BP1 (Ciccosanti et al., 2021). SARS-CoV-2 infection inhibits SG formation, which releases DDX3 to promote viral translation and replication (Ciccosanti et al., 2021).

### 5.3 DDX3X regulates HIV-1 mRNA translation

HIV-1 is a well-studied example where DDX3X contributes to viral translation through cap-dependent and independent mechanisms and its ability to form RNP granules. DDX3X is an essential host factor for HIV-1 replication (Yedavalli et al., 2004) and its function in translation likely underpins this requirement for DDX3X. The 5′UTR of the HIV-1 genome is a well-characterised viral RNA target of DDX3X, where it strongly binds to the stem-loop TAR motif located at the very 5′ end of the genomic RNA, although it can bind to additional downstream stem loops also (Soto-Rifo et al., 2012; Soto-Rifo et al., 2013). Deletion of this motif or insertion of an upstream unstructured sequence removed dependency on DDX3X for translation, suggesting that it uses its helicase activity to resolve the cap-proximal secondary structures and thereby makes the 5′ cap available for 43S PIC binding (Soto-Rifo et al., 2012). The N-terminal domain of DDX3X may be involved in linking CRM-1-dependent nuclear export of unspliced HIV-1 gRNA with its translation and it mediates accumulation in cytoplasmic granules (Frohlich et al., 2016). These granules contain PABP and eIF4G, but not eIF4E, suggesting the presence of a novel DDX3X-containing cap-binding complex which supports HIV-1 gRNA expression (Soto-Rifo et al., 2012; Soto-Rifo et al., 2013). In addition to supporting cap-dependent translation of the HIV-1 gRNA, DDX3X also supports its cap-independent translation *via* an IRES located in the 5′ Long Terminal Repeat region (Liu et al., 2011). DDX3X also enhances polysome occupancy of Tat and Rev mRNAs, suggesting that it promotes their translation (Lai et al., 2013). Expression of HIV-1 Tat conversely enhances expression of DDX3X, and both proteins physically interact, with DDX3X being required for recruitment of Tat to cytoplasmic granules and polysomes with viral RNA (Lai et al., 2013). Overall, HIV-1 appears to target DDX3X throughout infection to mediate various processes essential for the viral life cycle, employing its RNA helicase activity, interactions with eIFs, and possibly its propensity to form phase-separated RNP granules.

In summary, the abilities of DDX3X to resolve RNA secondary structures, interact with the translation machinery, participate in RNA granule formation and contribute to non-canonical translation initiation make it a prime target for exploitation by a diverse range of viruses. Targeting DDX3X with inhibitors as a form of broad-spectrum anti-viral therapy holds promise, however one will need to consider its diverse range of functions and how inhibiting these affects both viral replication and host cell translation.

## 6 Conclusion

An intense research effort has been made in recent years to identify DDX3X’s physiological mRNA targets in different cell types, driven by a desire to understand pathophysiology of the neurodevelopmental condition DDX3X syndrome and by the prospect that DDX3X inhibitors could serve as anti-viral and anti-cancer drugs. This has significantly increased knowledge about DDX3X’s role in translation regulation, yet many open questions remain: Through detailed analysis of known DDX3X binding targets, is it possible to identify determinants of DDX3X binding and translational regulation? How does DDX3X dynamically regulate translation in the context of LLP-separated RNA granules, not all of which might be conventional SGs? The mechanisms by which DDX3X regulates translation are also not fully clear, as we discussed in Section 3. Which step(s) in translation (pre-)initiation does DDX3X regulate and is this target-specific? Can DDX3X also negatively regulate translation of mRNAs aside from its role in SG formation, and if so, how? How does DDX3X interact with other RBPs to regulate translation of specific mRNA subsets? What are the consequences of DDX3X manipulation by chemical inhibitors or post-translational modifications for translation of specific mRNA target subsets? Answering this last question is particularly important as we continue to explore DDX3X’s suitability as a drug target. Undoubtedly, as research interest in DDX3X continues to grow and the RNP field is seeing development of many new methodologies to analyse translation, these questions will be answered in the next few years and hopefully allow identification of strategies for manipulating DDX3X function safely in a therapeutic context.

## Glossary

DDX3X: Dead-box protein 3X
gRNA: genomic RNA
eIF: eukaryotic Initiation Factor
RBP: RNA-Binding Protein
CLIP-seq: Cross-Linking and Immunoprecipitation + RNAseq
UTR: Untranslated Region
RAC1: Ras-related C3 botulinum toxin substrate 1
CERT: Cysteine-Enriched Regulator of Translation
G4: G-quadruplex
hnRNPK: heterogenous nuclear Ribonucleoprotein K
SRP: signal recognition particle
CBC: Cap-binding complex
CBP: Cap-binding protein
NMD: Nonsense-Mediated Decay
PABP: Poly-A Binding Protein
PIC: Pre-Initiation Complex
TOR: Target of Rapamycin
SRS: Small Ribosomal Subunit
NDUFA1: NADH dehydrogenase [ubiquinone] 1 alpha subcomplex subunit 1
IL: Interleukin
LRS: Large Ribosomal Subunit
RPL13: Ribosomal Protein L13
ODC1: Orthinine Decarboxylase 1
MITF-M: Microphthalmia-associated transcription factor
AR: Androgen Receptor
STAT1: Signal transducer and activator of translation 1
IFNß: Interferon ß
IRES: Internal Ribosome Entry Site
uORF: Upstream Open Reading Frame
ISR: Integrated stress response
SG: Stress granule
LLPS: Liquid-Liquid Phase Separation
CITE: Cap-independent translation site
FXR1: Fragile X mental retardation syndrome-related protein 1
ATF: activating transcription factor
CEBPA: CCAAT/enhancer-binding protein alpha
ETV1: ETS translocation variant 1
G3BP1: Ras GTPase-activating protein-binding protein 1
FMDV: Foot and Mouth Disease Virus
EMCV: encehalomyocarditis virus.
